# A Highly Sensitive Method to Detect Avocado Sunblotch Viroid for the Maintenance of Infection-Free Avocado Germplasm Collections

**DOI:** 10.3390/v11060512

**Published:** 2019-06-04

**Authors:** David N. Kuhn, Barbie Freeman, Andrew Geering, Alan H. Chambers

**Affiliations:** 1 Subtropical Horticultural Research Station, Agricultural Research Service, United States Department of Agriculture, Miami, FL 33158, USA; David.Kuhn@ars.usda.gov (D.N.K.); Barbara.Freeman@ars.usda.gov (B.F.); 2 Centre for Horticulture Science, Queensland Alliance for Agriculture and Food Innovation, The University of Queensland, St Lucia, QLD 4072, Australia; a.geering@uq.edu.au; 3 Tropical Research and Education Center, University of Florida, Homestead, FL 33031, USA

**Keywords:** ASBVd, RT-PCR, germplasm curation, viroid

## Abstract

The United States Department of Agriculture (USDA) Agricultural Research Service (ARS) Subtropical Horticulture Research Station (SHRS) in Miami, FL holds a large germplasm collection of avocado (*Persea americana*). The recent threat of infection by laurel wilt has encouraged the creation of a backup collection at a disease-free site. Creating the backup collection is complicated by infection of some trees in the germplasm collection with avocado sunblotch viroid (ASBVd). Infected trees are frequently asymptomatic, necessitating the use of a molecular diagnostic assay. Although a reverse-transcription based assay already exists and has been used to assay all germplasm at the station, some trees showed inconsistent results. We have developed a more sensitive and specific assay involving pre-amplification of the entire viroid cDNA followed by detection using real-time PCR and a TaqMan assay. A second screening of all germplasm identified additional ASBVd -infected trees and allowed us to confidently remove these trees from the station. This method enables avocado germplasm curators to proceed with the creation of a viroid-free backup collection.

## 1. Introduction

Avocado sunblotch viroid (ASBVd) is a single-stranded circular RNA molecule of 247 nucleotides (reference sequence variant GenBank J02020.1) that is confined in nature to avocado (*Persea americana*) [1]. Symptomatic and asymptomatic strains are recognized, although even the apparently asymptomatic strains are associated with yield loss [2]. The most characteristic symptom of sunblotch disease is sunken scars on the fruit surface, which may be pink or yellow depending on the color of the skin at maturity. ASBVd is transmissible through seed and mechanical damage, but has no insect vectors. Precautions need to be taken during propagation to prevent infection as rates of seed transmission are high at 86%–100%, and ASBVd can also be introduced through the use of infected budwood [3]. Rates of spread in the field are low and based on patterns of infection, it is suspected that ASBVd spreads in the field by root grafting. Pruning could potentially transmit the viroid but the efficiency of this mode of transmission is thought to be low [3]. Pollen transmission does occur, but this only results in infection of the seed and not the mother plant [4]. There are no known methods to cure trees of infection and even micrografting or somatic embryogenesis does not eliminate ASBVd [5,6]. Trees must therefore be rogued to prevent infection of neighboring trees.

The USDA-ARS Subtropical Horticultural Research Station (SHRS) in Miami, Miami-Dade County, Florida is a subtropical germplasm repository. As part of the National Germplasm Repository, SHRS distributes germplasm material through the GRINGlobal (www.grin-global.org). The SHRS repository is a living collection of subtropical and tropical species that have recalcitrant seeds that cannot be stored. Included in the collection are at least 270 unique accessions of avocado. Detection of ASBVd in the SHRS avocado collection ranged from 18%–21% of the trees sampled [7,8,9].

Avocado is the second most important fruit tree crop in Florida after citrus, with the majority produced in Miami-Dade County. Laurel wilt, a lethal fungal disease caused by *Raffaelea lauricola* and vectored by the red bay ambrosia beetle (*Xyleborus glabratus*), has been a problem in orchards in Miami-Dade since 2011 [10], but has not yet been observed at the SHRS. Due to the serious threat posed by this disease, a backup collection of the SHRS avocado germplasm is needed at an alternate site that is free of laurel wilt. However, ASBVd-infected accessions cannot be moved in order to prevent the spread of the viroid pathogen. In addition, for some SHRS germplasm trees, results of a reverse transcriptase PCR (RT-PCR) assay [11] were not consistent over years, suggesting that a more sensitive assay was needed to provide certainty that a tree was not infected prior to distribution to or inclusion in the backup collection.

The most sensitive assays for ASBVd are based on RT-PCR. Previously, the detection of the amplified fragment was by polyacrylamide gel electrophoresis or capillary electrophoresis [11,12,13]. Recently, a qPCR assay using real-time PCR of the reverse-transcribed viroid RNA has been developed using SYBR-Green. [14]. The SYBR-Green assay is reported to be ~100x more sensitive than previous RT-PCR methods, but is, therefore, also more sensitive to cross contamination during sampling. We describe here a method using nested primers for specific pre-amplification of the ASBVd viroid, specific detection of the viroid using a TaqMan gene expression assay, and quantitation by real-time PCR. This assay is sensitive enough to accurately detect a 1:10^7^ dilution of viroid RNA from a single infected tree and allows for confidence in detecting a single infected tree in pools of samples of eight trees. Using this assay, we identified and removed all infected trees from SHRS, which has allowed us to confidently create a backup collection and distribute viroid-free material.

## 2. Materials and Methods

### 2.1. Avocado Trees

Accession details for avocado trees used in experiments are described in Table 1. A full list of cultivar names is provided in Supplementary Table S1.

### 2.2. Viroid Indexing

#### 2.2.1. Sampling

For routine viroid indexing, one leaf from each of six positions on the tree (north, south, east, west, top north and top south) was collected and placed into a pre-labeled bag. Leaves from eight trees were combined in a single bag when pooling samples. To avoid cross-contamination of the viroid, tasks were divided, with one person collecting the samples and a second responsible for record-keeping and opening the bag to receive the leaves. The person sampling the leaves had gloved hands that were wiped with a 20% solution of commercial bleach after each tree and cutting tools were decontaminated in the same way. Leaves that were too old, damaged, or too young and thin were avoided. The decontamination process was repeated between each leaf sample when examining the distribution of the viroid within a tree at six positions (north, south, east, west, top north, and top south). A detailed leaf collection protocol is available in Supplementary Methods S3. One negative control sample, from a tree that had never tested positive over years of testing [8,9,15], was collected every 17 samples and was included on each extraction plate. Samples were stored in an ice cooler or at 4 °C until extraction.

#### 2.2.2. Tissue Disruption

Samples were processed within 48 h after collection by either manually dicing interveinal leaf tissue into 1 mm wide squares (~100 mg) using disposable blades and cleaning surfaces with 20% bleach between samples or by taking a leaf disc using a PlantTrak Lx Benchtop Plant Sampling & Barcoding by Brooks Life Sciences with a 3 mm punch head. The punch head was sterilized between samples by punching a Whatman PlantSaver FTA Card (WB120065, Whatman, GE Life Sciences, Pittsburgh, PA, USA) soaked in 20% bleach ten times and wiping the punch head and PlantTrak surface with a disposable paper towel with 20% bleach. The leaf tissue and a one 1/52’’ metal grinding bead were then added to individual wells of a 96 deep well plate in an arrangement where no samples were placed next to each other to avoid cross contamination. Samples were stored at −80 °C if RNA extractions were not done immediately. 

### 2.3. RNA Extraction

RNA was extracted using a modified method of Ainsworth [16]. Five hundred μL of extraction buffer with 0.16% (*w*/*v*) dithiothreitol was added to each 1.2 mL well, which was capped and wrapped in parafilm to avoid leakage. Each 96 well plate was ground in a Genogrinder 2000 (SPEX SamplePrep, Metuchen, NJ, USA) for 2 min at 2000 strokes per minute, centrifuged at 1000× *g* for 30 s, and the process repeated at least one more time until no large leaf pieces were visible. Plates were then centrifuged at 6100× *g* for 10 min at room temperature and the supernatant transferred to a new 1.5 mL tube, removing caps one sample at a time and washing gloves with bleach between samples. After adding a 1/3 volume of 8 M lithium chloride (LiCl), the samples were vortexed and left to incubate in an ice water bath placed in a 4 °C refrigerator overnight. After centrifugation at 10,000× *g* for 10 min at 4 °C, the supernatant was removed and pellets were resuspended in 100 µL of 2 M LiCl by flicking and pulse vortexing. The centrifugation and resuspension steps were repeated twice more, but after the final centrifugation, the pellets were resuspended in 50 µL of deionized, diethyl pyrocarbonate (DEPC)-treated water and the samples sat at room temperature for 10 min. One-tenth of a volume of 3 M sodium acetate and 2.5 volumes of 100% ethanol were added and samples were left overnight −20 °C. Samples were then centrifuged at 15,000× *g* for 10 min at 4 °C and the supernatant was removed. Pellets were rinsed with 100 µL cold 70% ethanol, mixed by flicking, and the centrifuge and 70% ethanol extraction were repeated. Samples were then dried for 5 min in a vacuum desiccator. Pellets were resuspended in 20 µL DEPC water. Samples were vortexed and allowed to incubate for at least 10 min at room temperature before quantification with a NanoDrop 2000 spectrophotometer (ThermoFisher Scientific, Waltham, MA, USA). Samples were normalized to 100 ng/μL prior to analysis by RT-PCR.

### 2.4. Primers and Probes

The primers for the control target, glyceraldehyde phosphate dehydrogenase (GAPDH), were designed across exon junctions to only amplify cDNA (Table 2). Primer and probe information for all other targets is provided in Table 2.

### 2.5. Reverse Transcription and Pre-amplification

GAPDH was used as the positive internal control [17] for the RNA extraction, reverse transcription (RT), pre-amplification, and TaqMan assay. Reverse transcription reactions were performed either as separate ASBVd and GAPDH reactions, resulting in an ASBVd cDNA sample and a GAPDH cDNA sample for each RNA extraction, or with the ASBVd and GAPDH primers multiplexed in one reaction. Reverse transcription reactions were performed on the avocado leaf samples using the High-Capacity cDNA Archive Kit from Applied Biosystems (ThermoFisher Scientific, 4368813) with modifications. Reactions were done in 20 µL volume with 10 µL master mix and 10 µL RNA sample at 100 ng/µL for a total of 1 µg RNA. Forward and reverse gene-specific primers (SB1-F1 and SB1-R1 for ASBVd, and GAPTM-F1 and GAPTM-R1new for GAPDH, see Table 2) at a final concentration of 0.5 µM were used instead of random primers, one set each for separate RT reactions and both sets for the RT multiplex. A no template control (NTC) was included for each primer pair. Reactions were run at 25 °C for 10 min, 37 °C for 120 min, 85 °C for 5 min, and held at 4 °C. Pre-amplification reactions consisted of 20 µL reactions including 1× buffer (M0270L, New England Biolabs, Ipswich, MA, USA), 0.2 µM dNTPs, 0.3 µM each forward and reverse primers (same gene specific primers as reverse transcription reactions), 0.8 units of *Taq* polymerase, and 2 µL of reverse transcription product. One NTC was included with each pre-amplification reaction plate. Reactions were run at 94 °C for 2 min, 10 cycles of 94 °C for 30 s, 60 °C for one minute, and 72 °C for one minute, then 49 °C for one minute, 72 °C for 5 min, and a hold at 4 °C. 

### 2.6. TaqMan Real-Time PCR Assays

TaqMan assays were performed on Fluidigm BioMark (Fluidigm, San Francisco, CA, USA) platform on a 192.24 gene expression chip. The assay mix included final concentrations of 0.6× Fluidigm 2× assay loading reagent (Fluidigm 100-76116), 5.4 µM each forward and reverse primers, and 1.2 µM probe in a total of 5 µL. Twenty ASBVd and 4 GAPDH assays were run on each chip, providing 20 ASBVd and 4 GAPDH replicate reactions for each sample, including NTC for each chip. The sample mix included 0.12× TaqMan 2× gene expression master mix (ThermoFisher Scientific, 4369514), 0.12× Fluidigm 20× GE sample loading reagent (Fluidigm, 100-7610), 1 µL ASBVd pre-amplification product, and 0.35 µL GAPDH cDNA in a total of 3 µL. The gene expression chip was prepared following the Fluidigm manufacturer’s instructions. All NTCs from reverse transcription and pre-amplification reactions were also run on both TaqMan assay platforms to be able to determine that each step was free of contamination. A positive signal was determined if the cycle threshold (CT) values for all replications of the sample were lower than the NTC CT value. If all replications did not show a CT value lower than the NTC, or if the CT value was over 30, that sample was rerun. If rerun samples showed consistent values over 30, but lower than the NTC, the CT value of that sample was considered positive.

### 2.7. Roguing Infected Trees

Trees testing positive for ASBVd were rogued. Infected trees were either knocked over or dug up depending on size. The resulting debris were collected and discarded offsite.

### 2.8. Creation of a Backup Germplasm Collection

Budwood from viroid-free trees was couriered to the USDA-ARS Foreign Disease-Weed Science Research Unit in Ft. Detrick, MD (FDWSRU) and grafted to viroid-free rootstock. After at least one year’s growth in the greenhouse, two leaves from the grafted scions were retested for ASBVd infection. Budwood from viroid-free trees were shipped to the USDA-ARS Pacific Basin Agricultural Research Center in Hilo, HI (PBARC) for backup collection maintenance. Grafted trees at PBARC were also retested for ASBVd infection before planting.

## 3. Results

### 3.1. Assay Design and Testing

**Figure 1:**
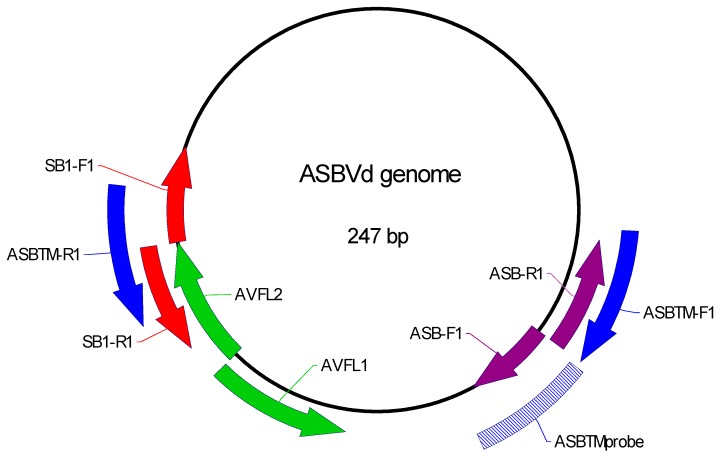
Primers tested for assay design. Primer locations on the ASBVd genome, including primer sequences, are listed in Table 2.

Four sets of primers for detection of the ASBVd molecule have already been designed (Table 1, Figure 1). Three of these amplify the entire ASBVd molecule, as shown in Figure 1: the red (SB1-F1 and SB1-R1, Semancik) [13], the purple (ASB-F1 and ASB-R1, Schnell et al.) [11], and the green (AVFL1 and AVFL2, Randles et al.) [19]. The blue primers (ASBTM-F1 and ASBTM-R1 [17] amplify a section of the ASBVd molecule which was used to design the TaqMan probe. Initial analysis of the red, purple, and green primer sets revealed that the red primers amplified the positive control tree with the most consistent signal (average CT 16.01, standard deviation 0.6, average tm 76.28, standard deviation 0.1) and never amplified the no-template control. Further assay development used the red primers only. 

The objective of this study was to increase the sensitivity of the RT-PCR assay by pre-amplifying the entire ASBVd molecule with one set of primers and then assaying the amplified molecule with another set of primers that targeted only a section of the ASBVd molecule amplified by AVLF1 and ASBTM1. We tested detection of the smaller amplicon using a TaqMan assay for a probe complementary to a sequence in the smaller amplicon (Figure 1). The red primers were used for pre-amplification. As the reverse blue primer sequence ASBTM-R1 overlapped the ends of the red primers, the green primer AVFL1 was used with the forward blue (ASBTM-F1) and probe (ASBTM) for the TaqMan assay.

### 3.2. Sensitivity Determination

The sensitivity of this assay was tested using serial dilutions from a positive control sample. A tree that had always tested positive for ASBVd (“Aycock Red” No. 19, Table S1) was used as the ASBVd positive control in the absence of a purified source of viroid. A tree that had always tested negative for ASBVd (“Collinred B”, Table S1) was used as a negative control. The following serial dilutions were made from ASBVd cDNA of the entire molecule from the ASBVd positive control (PC-ASBVd cDNA) using water as the diluent: 1:10^3^, 1:10^4^, 1:10^5^, 1:10^6^, 1:10^7^, 1:10^8^, and 1:10^9^. The ASBVd negative control cDNA (N4-ASBVd cDNA) was cDNA from a reverse transcription reaction with ASBVd primers on the negative control tree. The ASBVd negative control cDNA was used for background in the pre-amplification reaction to simulate the normal assay conditions (undiluted cDNA) for each dilution. The pre-amplification product was not diluted. Twelve replicates of each dilution were run on a 192.24 GE Fluidigm chip on the BioMark, with 12 ASBVd assays and four GAPDH assays, providing 144 replicate reactions for each dilution. It was determined that an ASBVd positive signal could be detected reliably in the real-time TaqMan PCR assay on the Fluidigm BioMark at a dilution of 10^7^ (Table 3).

### 3.3. Distribution of ASBVd in A Single Tree

Great variation in viroid titer was observed in experiments to determine the distribution of the viroid in a tree (Table 4). None of the trees tested for titer distribution showed visual symptoms of the viroid. Average CT values across all sampled trees ranged from 7.3 to no amplification, and from 9 to no amplification within a single tree (Honalindo 1).

### 3.4. Pooling Samples and Multiplex of ASBVd and GAPDH

Three separate RNA extractions were isolated from three separate pools consisting of 49 leaf discs from the negative control tree and one leaf disc from the positive control tree to investigate assay sensitivity for detecting lower levels of ASBVd contamination in pooled samples. A separate collection was made for each pool making biological replicates. In addition, 1:50 and 1:100 dilutions of samples from FDWSRU were assayed on the Fluidigm BioMark. The 49:1 negative to positive RNA showed clear positive results (CT under 10, Table S2) and the majority of the FDWSRU sample dilutions showed a positive signal where CT values for all replicates were lower than 30.

The pooled and diluted FDWSRU samples were also run with the ASBVd and GAPDH multiplexed in the reverse transcription. After determining that using the control (GAPDH) internally in the ASBVd RT reaction resulted in an ~26% decrease in sensitivity (Table S2), two separate reverse transcription reactions were performed on the avocado leaf samples, and samples were then assayed with separate ASBVd and GAPDH cDNA. Furthermore, the fluorescence for the GAPDH when it was pre-amplified was often so high it would increase the gain or sensitivity of the Fludigm BioMark and mask the ASBVd signal. Therefore, samples were run on the TaqMan assay with ASBVd pre-amplification product and a separate GAPDH cDNA. The number of cycles in pre-amplification was also optimized to minimize background or over-fluorescence, especially in samples with high levels of ASBVd contamination.

### 3.5. Application

Leaves from grafted trees of previously negative SHRS avocado trees in FDWSRU were re-tested with the current assay. If any of the trees in FDWSRU tested positive, the source tree at SHRS was tested with the TaqMan assay (Table S1). These trees at the SHRS were also used to test leaves from different locations on the tree (Table 4). All grafted tree samples at FDWSRU were re-tested with the TaqMan assay before being sent to PBARC. Budwood sent from FDWSRU was grafted at PBARC Hawaii and all trees were tested with the TaqMan ASBVd assay before they were planted in the field. ASBVd positive grafts were detected at FDWSRU from ASBVd negative genotypes at SHRS. 

**Figure 2:**
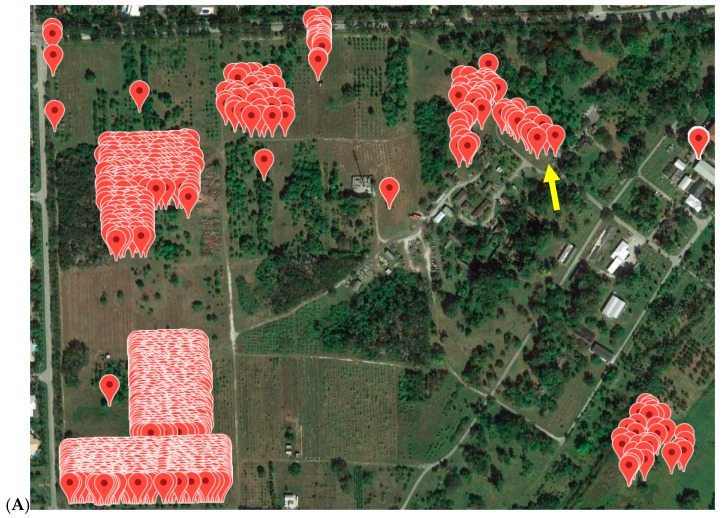

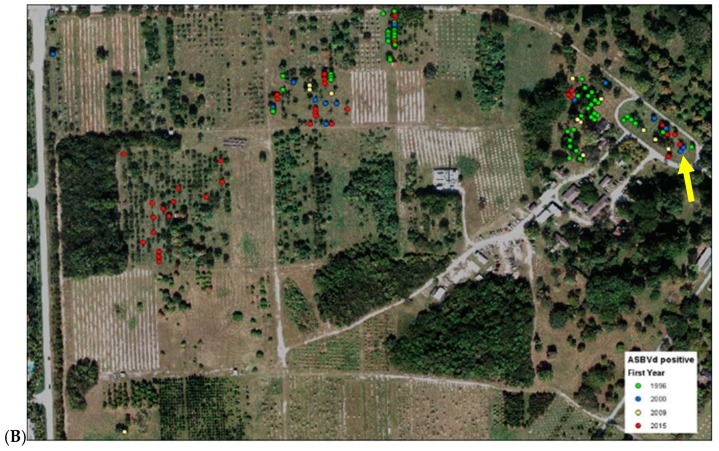
(**A**) Location of all avocado trees on the SHRS station and (**B**) location and first detection of ASBVd positive avocado trees on the SHRS station. Yellow arrow points to “Avocado Circle”.

All avocado trees in the germplasm collection at the SHRS were tested with the TaqMan ASBVd assay between 2014 and 2016. Trees were first sampled in pools of 8, then all trees in pools that showed positive results were tested separately. If a tree was consistently positive over two or more previous testing years, or if a tree showed phenotypic signs of ASBVd infection, they were considered positive and were not tested again. Out of 435 clonal avocado trees, 113 tested positive (26%, Table S1). A random sample of 50 trees, pooled in groups of eight, was tested from the Florida avocado mapping population. All these trees were negative. The origin of the ASBVd infection was in “Avocado Circle”, a collection of cultivars that showed signs of ASBVd infection since 1992 (Figure 2). Almost all trees located in this circle were infected. The majority of an older avocado collection located in close proximity to Avocado circle were also infected. Infected trees were removed as described in Materials and Methods. 

## 4. Discussion

The mission of the SHRS includes the maintenance, curation, and distribution of disease-free germplasm through the GRINGlobal system. The SHRS houses many collections of tropical/subtropical species, with avocado and mango being two of the more important collections. As seeds of these trees cannot be stored, nor would they represent the genetically identical cultivars in the collection, we maintain living collections in the field and distribute budwood of the accessions. Curating such collections is complicated by both abiotic (hurricanes) and biotic (disease) stresses. We have been aware, since 1992, that avocado trees in our collection are infected with ASBVd, which prompted our development of an RT-PCR assay to detect the viroid in symptomless trees [11]. Nonetheless, some trees were found to be positive in one year’s assay and negative in another. When it became clear that ASBVd was spreading at the station [7], we realized the need for a more sensitive assay to confidently identify all infected trees. The issue of infected trees became even more pressing when laurel wilt was discovered in Miami-Dade County in 2011 [10]. To preserve the germplasm collection, prophylactic treatment with fungicide was begun and a backup collection at PBARC was planned. Hawaii has an avocado production industry and no ASBVd has been detected in Hawaii. Thus, to create a backup germplasm collection in Hawaii to avoid infection with laurel wilt, a highly sensitive ASBVd assay was needed to confidently identify viroid-free trees prior to shipping to Hawaii.

Our assay is an improvement on previous RT-PCR and real-time RT-PCR assays as it utilizes a pre-amplification process for increased sensitivity and a probe detection of an ASBVd region with increased specificity. Comparison of our assay with other published assays is difficult as each research group has used a different standard ASBVd (plasmid DNA, transcribed plasmid DNA) to estimate sensitivity. Our goal was to develop an assay sensitive enough to guarantee no false negatives and based our sensitivity studies on known infected trees, known uninfected trees, and trees that had shown different results over different years.

We also addressed the problem of cross-contamination of samples during collection, processing, and assay. Collection tools and leaf cutting tools are easily and permanently contaminated with viroid RNA that causes many false positives. In addition, as our method became more sensitive, the potential for cross-contamination simply from opening tube caps was also observed and led to the contamination prevention protocols described in Material and Methods. False positives are of major concern in curating a germplasm collection, especially when the only prophylactic method is tree removal. Additionally, ASBVd is transmissible through pollen, and seed propagated ‘Lula’ rootstock from SHRS could have contributed to ASBVd positive results in grafted plants from ASBVd negative budwood. This indicates the need for caution to ensure disease-free rootstocks are used when establishing backup collections for avocado.

SHRS has more than 2000 avocado trees including research populations. Attempting to assay so many trees on an annual or biannual basis is beyond our resources. We focused first on removing ASBVd infected trees from the germplasm collection. Then, we improved the efficiency of our method by determining that we could accurately identify viroid infection by sampling four leaves from the cardinal points and two leaves from the top of each tree in the field. Although differences were seen in viroid amount among the six leaves, the assay was sensitive enough to accurately determine infection from this six-leaf assay. We also demonstrated that viroid from a single leaf disk from an infected tree could be detected in the presence of a pool of 49 leaf disks from an uninfected tree. Since we harvested six leaves from each tree, this meant we could pool the leaves from eight trees for each assay. If the pooled assay was positive for viroid, we would then assay each tree in the pool individually. Finally, we are in the process of collecting a random sample of 48 trees (six pools of eight trees) per field at the station using the same method as above. Our initial random samples showed no viroid infection of any of the 1500 trees in our research populations. Assaying random samples from each field makes it possible to assay the entire population of avocado trees on the station each year.

It is always difficult to destroy trees in a germplasm collection, especially if this leads to the loss of all representatives of a cultivar. However, having viroid infected trees on the station prevents us from distributing material and in creating a backup collection. Since all the infected trees have been removed, we have completed our backup collection at PBARC and are able to confidently distribute avocado budwood upon request. Any plot that contained an infected avocado tree will not be used to replant an avocado as persistence of viroid RNA in the soil is too great a risk. 

## Figures and Tables

**Table 1:** Avocado trees that were tested for avocado sunblotch viroid (ASBVd).

Population	Original Source ^1^	Current Location ^1^	Number Individuals Sampled	Number Individuals with Inconsistent Results from Previous Assay
Backup germplasm collection	SHRS ARS	FDWSRU ARS Ft. Detrick, Maryland	131	10
Avocado germplasm collection	SHRS ARS	SHRS ARS Miami, Florida	383	50
Avocado mapping population ^2^	SHRS ARS	SHRS ARS Miami, Florida	50	NPA ^3^
Backup germplasm collection	SHRS ARS	PBARC ARS Hilo, Hawaii	102	NPA

^1^ Abbreviations are: SHRS ARS, Subtropical Horticultural Research Station; FDWSRU ARS, Foreign Disease-Weed Science Research Unit; and PBARC ARS, US Pacific Basin Agricultural Research Center; ^2^ See Olano et al. [15] for details of mapping population; ^3^ NPA, not previously assayed.

**Table 2:** Primers and probes for ASBVd and glyceraldehyde phosphate dehydrogenase (GAPDH) control for TaqMan and SYBR-green assays. Primer name and sequences are shown with base pair position in the ASBVd genome, detection methods used in this study, and target references.

Primer Name	Primer Color in Figure 1	Primer Sequence (5′→ 3′)	Position in ASBVd Genome	Reaction	Reference
GAPTM-F1		TGGAGTGGACAGTGGTCATCAG		RT and TaqMan	Geering, ADW [17]
GAPTM-R1new		CCCATTGGCCAAGGTGATC		RT and TaqMan	This study
GAPDHTM-probe		[VIC]-CCCTCAACAATGCC-MGBNFQ		TaqMan	Geering, ADW [17]
SB1-F1	Red	TGGGAAGAACACTGATGAG	180–198	RT and preAmp	Semancik, JS. [13]
SB1-R1	Red	TCTTTCCCTGAAGAGACGA	179–161	RT and preAmp	Semancik, JS. [13]
ASBTM-F1	Blue	TTCCGACTCTGAGTTTCGACTT	66–87	TaqMan	Geering, ADW [17]
AVFL1	Green	CAAGAGATTGAAGACGAGTGAACTA	179–155	TaqMan	Randles et al. [18]
ASBTM-probe	Hatched	[6FAM]TTCCGACTCTGAGTTTCGACTT-MGBNFQ	89–107	TaqMan	Geering, ADW [17]
ASB-F1	Purple	GTGAGAGAAGGAGGAGT	88–104		Schnell et al. [11]
ASB-R1	Purple	AAGTCGAAACTCAGAGTCGG	87–68		Schnell et al. [11]
AVFL2	Green	ATCACTTCGTCTCTTCAGGGAAAGA	130–154		Randles et al. [18]
ASBTM-R1	Blue	GTTCTTCCCATCTTTCCCTGA	189–168		Geering, ADW [17]

**Table 3:** ASBVd sensitivity test on Fluidigm BioMark Real-Time assay. Positive control samples were prepared by manual cutting. Dilution, CT average, and CT standard deviation are shown. “No amp” indicates no amplification.

Dilution	CT avg	CT SD
1:10^3^	17.6	2.0
1:10^4^	24.3	1.9
1:10^5^	25.5	1.4
1:10^6^	29.5	2.4
1:10^7^	31.1	2.4
1:10^8^	No amp	0.0
1:10^9^	No amp	0.0

**Table 4:** ASBVd signal variation on different locations of the tree. Samples prepared by manual cutting. Higher viroid abundance is shown by lower average CT values. “No amp” indicates no amplification. Values for ASBVd screening of trees grafted from the individual at FDWSRU are included, (NG = tree not grafted).

Sample ID			Avg CT						Avg CT all Locations *	FDWSRU Graft av CT
Cultivar	Location (SHRS)	Genotype ID	East	North	South	Top North	Top South	West		
AYCOCK RED NO. 19	W3-1-08-01	PC	22.3	9.3	7.3	8.4	8.2	8.0	10.6	NG
CELLON’S HAWAII SDLG	W3-1-02-01	CFN01	26.9	25.0	39.4	33.8	35.6	No amp	33.6	NG
SHARWIL	WA2-12-37	CFN19	26.9	25.0	22.9	23.9	25.2	21.9	24.3	NG
JOSE ANTONIO	WA2-20-32.2	CFN22	No amp	No amp	No amp	17.9	24.0	No amp	34.3	NG
R06-T05	WB4-02-13	FtD3	No amp	No amp	18.0	No amp	No amp	11.5	32.2	18.3
LA PISCINA	WB3-18-08	FtD30	22.3	21.8	23.9	No amp	24.8	34.0	28.0	18.9
DARIAN	WB3-19-11	FtD36	20.0	22.3	No amp	28.2	17.3	No amp	28.3	11.0
SEMIL 43	WA2-13-41	FtD96	22.2	20.5	22.2	16.4	17.3	12.7	18.6	29.1
P. NUBIGENA	WB3-10-03	FtD97	16.2	23.3	24.8	No amp	22.1	20.7	24.7	8.2
PIC 9615	WB3-13-10	FtD105	19.7	No amp	No amp	No amp	13.4	No amp	32.8	16.2
PINKERTON	WB4-09-01	FtD107	No amp	No amp	23.2	No amp	22.2	No amp	34.9	6.1
DADE SDLG	WB4-04-17	FtD110	No amp	18.5	38.3	No amp	No amp	No amp	36.8	7.8
HONALINDO 1	WB3-10-07	FtD117	19.2	21.6	No amp	9.5	9.0	No amp	23.5	8.8

* Real time assay runs for 40 cycles so “No amp” results were given a value of 41 for averaging.

## Supplementary Materials

The following are available online at https://www.mdpi.com/1999-4915/11/6/512/s1; Supplementary Table S1 with SHRS assay results, Supplementary Table S2 with ASBVd Fluidigm negative to positive pools, and Supplementary Methods S3 with detailed ASBVd assay leaf collection protocol.
