# Supplementary material for: A Highly Sensitive Method to Detect Avocado Sunblotch Viroid for the Maintenance of Infection-Free Avocado Germplasm Collections
Source: Viruses. 2019 Jun 4;11(6):512. doi: 10.3390/v11060512 (PMC6630647; doi:10.3390/v11060512)
Supplement: Supplementary file 1 [file viruses-11-00512-s001.zip › S3 ASBVd assay leaf collection protocol.docx]

**Supplementary Material S2**

**ASBVd assay leaf collection protocol**

Bring to the field: lots of gloves, 20% commercial bleach in a spray bottle, bucket, paper towels, cooler or box with ice packs, plastic or paper bags labeled with location, tree ID, and barcode, rubber bands, checklist, pencil, marker, extension clippers

Work in pairs of two people, have one person be the dirty: collect the leaves, bundle sample, and place in bag; and the other be the clean: determine tree directions with compass, open sample bag, check off leaves, close and place sample bag in cooler (steps marked with “clean” or “dirty” below).

When treating samples separately each bag represents one sample or individual tree, 6 leaves from each tree. When pooling samples each bag represents one sample pool, which includes 8 trees, 6 leaves from each tree (48 leaves total).

- Travel to the first tree on the collection list (Location) and find North side of tree based on a compass (clean).

- Put on gloves (dirty).

- Open corresponding sample bag or sample pool bag (see collection list) and place it or hold it where it is easy to place sample bundle inside, but where it will not be contaminated by leaves or you (clean).

- Snap off at the base of the stem **one** mature leaf from the North side of the tree. Try to get a green leaf, **not** red, too old, damaged, or young and thin. Also try to get them low enough on the tree to distinguish them from top of the tree samples (dirty).

- Repeat with the East, South, and West sides of the tree. Be sure to only collect **1** leaf from each side (dirty).

- Collect **one** leaf from as close to the top of the canopy as you can on the North side. If needed use extension clippers that have been sanitized with bleach (dunk in bucket of bleach solution or spray down with bleach solution, wipe away any plant pieces with a clean paper towel if applicable. Also, do not touch the handle of the clippers with contaminated gloves, rinse gloves in bleach first) (dirty).

- Collect **one** leaf from as close to the top of the canopy as you can on the South side. Always be sure to only collect **1** leaf from each side. Sanitize clippers with bleach (dirty).

- Carefully place sample bundle in sample bag for separate samples or if pooling samples bind the 6 leaves together with a rubber band (tight enough that they stay together but not so tight to damage the leaves) and place sample bundle in sample pool bag. Do not touch outside of bag with gloved hands or anything that has touched avocado parts (dirty).

- Thoroughly wash gloved hands and extension clipper with bleach (dunk in bleach bucket) (dirty).

- Close sample bag or sample pool bag with a rubber band or ziplock and place inside cooler or box. Do not touch the cooler or box with gloved hands or anything that has touched avocado parts (clean).

- Check off leaves collected on the collection list and record the date. Do not touch the list or pencils with gloved hands or anything that has touched avocado parts (clean).

- Travel to the next tree on the collection list and repeat all steps with the next sample. If pooling samples repeat until you have all 8 trees in the sample pool bag.

- Follow all sterilization procedures etc. to collect negative control (NC) samples.

Note: To determine presence of ASBVd from a single sampling site (north, south, etc.) an individual leaf is collected and placed in a labeled bag and tools and gloves are bleached before collection from the next position on the same tree.
